# Restriction on the local realism violation in three-qubit states and its relation with tripartite entanglement

**DOI:** 10.1038/s41598-018-30022-7

**Published:** 2018-08-17

**Authors:** Artur Barasiński

**Affiliations:** 1 RCPTM, Joint Laboratory of Optics of Palacký University and Institute of Physics of CAS, Faculty of Science, Palacký University, 17. Listopadu 12, 771 46 Olomouc, Czech Republic; 2 Institute of Physics, University of Zielona Góra, Z. Szafrana 4a, 65-516 Zielona Góra, Poland

## Abstract

Quantum entanglement and non-locality are two special aspects of quantum correlations. The relationship between multipartite entanglement and non-locality is at the root of the foundations of quantum mechanics but there is still no general quantitative theory. In order to address this issue we analyze the relationship between tripartite non-locality and tripartite entanglement measure, called the three-tangle. We describe the states which give the extremal quantum values of a Bell-type inequality for a given value of the tripartite entanglement. Moreover, we show that such extremal states can be reached if one introduced an appropriate order induced by the three-*π* entanglement measure. Finally, we derive an analytical expression relating tripartite entanglement to the maximal violations of the Bell-type inequalities.

## Introduction

Quantum entanglement and non-locality that is certified by the violation of Bell-type inequalities^1–4^, are two special aspects of quantum theory, which distinguish quantum from the classical world. Both quantities play a central role for numerous quantum information protocols, in particular in quantum computation, quantum communication and quantum cryptography^5,6^. However, the question if entanglement and/or non-locality are necessary resources for quantum protocols is still open.

It is long known that Bell inequality violations implies entanglement. However, the reverse implication is not true^7^. As it was proven recently^8^, for any number of parties *N* there exist states that do not violate any Bell inequality for genuine N-partite non-locality despite being genuinely N-partite entangled. This means that entanglement and non-locality are inequivalent and hence they are different resources. Understanding the relation between these resources is therefore one of the important problems of quantum theory from both fundamental and applied points of view. While such relation has been successfully studied for the case of two qubits^9,10^ and recently for pure two-qudit state^11^, very little is known in the multipartite scenario. It is because the multipartite scenario offers a richer variety of different types of entanglement and non-locality. For instance, a three-qubit state can either be completely separable, biseparable or tripartite entangled, Furthermore, there are two locally inequivalent classes of tripartite entanglement, namely the GHZ-class and the W-class^12,13^. None of the GHZ-class states can be transformed by local invertible operations into the W-class states and *vice versa*.

In this paper we investigate the problem of the restriction on the nonlocal correlations imposed on the tripartite qubit state with a given genuine tripartite entanglement (GTE). In other words, we ask, for a fixed Bell scenario, what is the best one can obtain optimizing over all possible quantum resources (states and measurements) preserving fixed entanglement properties^14–20^?

First attempts to determine the restriction on the nonlocal correlations for three-qubit states have been performed by Emary *et al*.^15^. It has been shown (in the frame of numerical and later analytical calculations^15,17^) that within the three-parameter pure GHZ subfamily |Φ〉 (discussed later) the upper and lower bounds of the maximal violation of the Bell-type inequalities for a given tripartite entanglement monotony (the three-tangle^21^) are provided by the maximal slice (MS) states^22^ and the generalized GHZ (GGHZ) states^12^, respectively. The Bell-type inequalities have been taken of the Mermin^23^ and Svetlichny^4^ form. However, for general three-qubit state |*ψ*〉 these results are no longer true^15^. Here, we establish the extremal quantum values of a Bell-type inequality in general case (henceforth referred to as the Tsirelson-like bound). As a primary entanglement measure we also use the three-tangle. Although, it should be mentioned that numerous approaches to define GTE measures have been proposed^24^, including the concepts of entropy vectors^25^, maximally entangled sets^26^ or source and target volumes of reachable states^27^. Our motivation arise from several reason. First, the three-tangle is directly related with the monogamy of entanglement which has been proven to be a universal feature of single-copy entanglement that is deeply rooted in the algebraic structure of quantum theory^28^. Monogamy of entanglement find an applications in many areas of physics, such as quantum information and the foundations of quantum information mechanics^29–32^, condensed-matter physics^33–35^, and even black-hole physics^36,37^. Furthermore, the three-tangle is the only independent SL-invariant tripartite entanglement monotony^38^, i.e. it is invariant under SL(2, ${\mathbb{C}}$) transformations on each qubit. Correspondingly, all pure three qubit states |*ψ*〉 with *τ*(*ψ*) = 0 and *τ*(*ψ*) ≠ 0 are locally equivalent to the W and GHZ state, respectively. Finally, the three-tangle is widely used in many quantum protocols, for instance, recently studied controlled teleportation and a minimal control power in controlled teleportation^39,40^.

In present work we show that the Tsirelson-like bound can be estimated by simultaneous use of two GTE monotonous, namely, the three-tangle and three-*π*^41^ which is a tripartite analog of the entanglement negativity^42–44^. For this purpose, we first present an extensive investigation of the quantitative relation between both GTE monotonous what is essentially important not only by fundamental implications^45–48^ but also by renewed interest in the GTE in terms of entanglement negativity in several contexts: the so-called “disentangling theorem”^49^, conformal field theory^50^ and topological order^51,52^, to name a few. Our results provide a motivation for further work on the understanding and physical interpretation of GTE for both the theoretical studies and practical applications^53^.

## Theory

In order to facilitate the discussion of our results, we first briefly describe two GTE monotonous (three-tangle and three-*π*) and tripartite nonolcality measurements.

### GTE monotonous

The three-tangle, *τ*, has been derived from the three-qubit monogamy relation discovered by Coffman, Kundu and Wootters (CKW)^21^ as a remanding entanglement that cannot be captured by the entanglement quantifiers of different reduced states of a composite quantum system (therefor, originally was called as the residual entanglement). For a tripartite pure state $|\psi \rangle ={\sum }_{m,n,p}\,{\mu }_{mnp}|mnp\rangle$ (hereafter the standard form) the three-tangle is written in the following form

1 $$\tau (\psi )={{\mathscr{C}}}_{A(BC)}^{2}-{{\mathscr{C}}}_{AB}^{2}-{{\mathscr{C}}}_{AC}^{2},$$

where ${{\mathscr{C}}}_{A(BC)}\equiv {\mathscr{C}}{(|\psi \rangle }_{A(BC)})$ represents the amount of bipartite entanglement between qubit A and the composite qubits BC quantified by the Wootters’ concurrence^54^. The remaining two terms, ${{\mathscr{C}}}_{AB}\equiv {\mathscr{C}}({\rho }_{AB})$ and ${{\mathscr{C}}}_{AC}\equiv {\mathscr{C}}({\rho }_{AC})$, stand for the concurrences of the appropriate two-qubit reduced states. It is noteworthy that the three-tangle is independent on the choice of the indices that enter in the considered bipartition. It is because *τ* is invariant under permutations of subsystem indices. However, this peculiar symmetry ends if we consider for more general cases^21^. The three-tangle has also been proposed for mixed states via a convex-roof extension^55^ of Eq. (1) on pure states

2 $$\tau (\rho )=\mathop{{\rm{\min }}}\limits_{\{{p}_{i},{\varphi }_{i}\}}\,\sum _{i}\,{p}_{i}\tau ({\varphi }_{i}),$$

where the minimum is taken over all convex decompositions $\rho ={\sum }_{i}\,{p}_{i}|{\varphi }_{i}\rangle \langle {\varphi }_{i}|$. Note that the general investigation of mixed-state three-tangle has been proven to be notoriously difficult^56,57^.

Later, Ou and Fan^41^ have shown that one can derive the analog of the CKW monogamy relation in terms of the entanglement negativity^42–44^ and hence an alternative GTE measures can be proposed as

3 $${\pi }_{A}(\rho )={{\mathscr{N}}}_{A(BC)}^{2}-{{\mathscr{N}}}_{AB}^{2}-{{\mathscr{N}}}_{AC}^{2},$$

where *π*_*A*_ denotes the tripartite entanglement with respect to the qubit A and ${\mathscr{N}}$ stands for entanglement negativity. The index *A* on the left-hand side comes from the fact that contrary to the three-tangle, here the permutation symmetry is broken even for N = 3. Therefore, the equation (3) can be written in the similar form with respect to qubits B (*π*_*B*_) and C (*π*_*C*_) taken as the focus, where in general *π*_*A*_ ≠ *π*_*B*_ ≠ *π*_*C*_. To make three-*π* invariant under permutations of the qubits the average of *π*_*A*_, *π*_*B*_ and *π*_*C*_ is taken^41^

4 $${\pi }_{ABC}(\rho )=\frac{1}{3}({\pi }_{A}(\rho )+{\pi }_{B}(\rho )+{\pi }_{C}(\rho )).$$

It should be mentioned that *π*_*ABC*_, in a form presented above, can be applied for both pure and mixed states. However, for higher-dimensional mixed states the entanglement negativity cannot distinguish separable from PPT bound entangled states^58^ i.e. a set of entangled states that cannot be distilled. In such cases, ${{\mathscr{N}}}_{i(jk)}$, where *i*, *j* and *k* are different from each other and *i*, *j*, *k* = {*A*, *B*, *C*}, may “underestimate” the amount of entanglement. Despite of that there is a large family of mixed entangled state for which negativity is sufficient to be entanglement measure and hence, the three-*π* can be used for such states.

### Non-locality measurements

The analysis of the Bell-type inequalities based on absolute local realism for three-qubit states is complicated by the need to distinguish between bipartite and tripartite non-locality. To overcome this problem we have used the Svetlichny inequality^4^ and (in order to confirm our predictions) the generalized Bell inequality based on the formalism proposed by Żukowski and Brucner^3^. An interesting difference between the two approaches has already been pointed out for instance in ref.^17^.

Suppose now that we have an ensemble of three spatially separated qubits, and the measurement ${V}_{A}=\overrightarrow{a}\cdot {\overrightarrow{\sigma }}_{A}$ or ${V}_{A}^{^{\prime} }=\overrightarrow{a}^{\prime} \cdot {\overrightarrow{\sigma }}_{A}$ on qubit *A*, ${V}_{B}=\overrightarrow{b}\cdot {\overrightarrow{\sigma }}_{B}$ or ${V}_{B}^{^{\prime} }=\overrightarrow{b}^{\prime} \cdot {\overrightarrow{\sigma }}_{B}$ on qubit *B*, ${V}_{C}=\overrightarrow{c}\cdot {\overrightarrow{\sigma }}_{C}$ or ${V}_{C}=\overrightarrow{c}^{\prime} \cdot {\overrightarrow{\sigma }}_{C}$ on qubit *C*. Here $\overrightarrow{a}$, $\overrightarrow{a}^{\prime}$, $\overrightarrow{b}$, $\overrightarrow{b}^{\prime}$ and $\overrightarrow{c}$, $\overrightarrow{c}^{\prime}$ are unit vectors and $\overrightarrow{\sigma }=\{{\sigma }_{x},{\sigma }_{y},{\sigma }_{z}\}$, where *σ*_*x*_, *σ*_*y*_ and *σ*_*z*_ are the Pauli operators associated with three orthogonal directions. If a theory is consistent with the hybrid nonlocal-local realism, then the expectation value for any three-qubit state is bounded by Svetlichny’s inequality

5 $$S(\rho )\equiv |{\rm{Tr}}(\hat{S}\rho )|\le 4,$$

where the Svetlichny operator is defined as $\hat{S}={V}_{A}({V}_{B}{V}_{D}+{V}_{B}^{^{\prime} }{V}_{D}^{^{\prime} })+{V}_{A}^{^{\prime} }({V}_{B}{V}_{D}^{^{\prime} }-{V}_{B}^{^{\prime} }{V}_{D})$ with ${V}_{D}={V}_{C}+{V}_{C}^{^{\prime} }$ and ${V}_{D}^{^{\prime} }={V}_{C}-{V}_{C}^{^{\prime} }$.

On the other hand, the Żukowski and Brucner proposal is as follow. Consider an arbitrary state *ρ* written as a tensor product of local Pauli operators in the form $\rho =\frac{1}{8}\,{\sum }_{\alpha ,\beta ,\gamma }\,{T}_{\alpha \beta \gamma }{\sigma }_{\alpha }{\sigma }_{\beta }{\sigma }_{\gamma }$, where *α*, *β*, *γ* = {0, *x*, *y*, *z*}, *σ*_0_ refers to the identity operator and *σ*_*x*_, *σ*_*y*_ and *σ*_*z*_ are the Pauli. The coefficients *T*_*αβγ*_ = tr(*ρ σ*_*α*_*σ*_*β*_*σ*_*γ*_) are the elements of the extended three-qubit correlation tensor $\hat{T}$. The necessary and sufficient condition for quantum state *ρ* to satisfy the generalized Bell inequalities is

6 $$T(\rho )\equiv \sum _{\alpha ,\beta ,\gamma }\,{g}_{\alpha }^{A}{g}_{\beta }^{B}{g}_{\gamma }^{C}|{T}_{\alpha \beta \gamma }|\le 1,$$

for any set of local coordinate systems of three observers and for any set of unit vectors ${\overrightarrow{g}}^{i}=({g}_{x}^{i},{g}_{y}^{i})$^3,59^. If the above relation is violated at least for one choice of local coordinate systems, no local realistic description of the tripartite correlation function is possible, in the case of any standard Bell experiment. Note that in Eq. (6) the sum is taken over any two orthogonal axes. To achieve this, one can choose, say, *x* and *y* directions and then perform arbitrary local transformations of the correlation tensor $\hat{T}$ (see refs^3,59,60^). In particular, one can express such local transformations by mean of Euler theorem. The inequality (6) can be further simplified by mean of the Cauchy inequality applied to the middle term. As a result, one obtains

7 $$T^{\prime} (\psi )\equiv \sum _{\alpha ,\beta ,\gamma }\,{T}_{\alpha \beta \gamma }^{2}\le 1,$$

which (in general) is just a sufficient condition for the existence of local description^3,59^.

We emphasize that throughout this paper the maximum expectation values of *T*, *T*′ and *S* (denoted as *T*_max_, ${T}_{{\rm{\max }}}^{^{\prime} }$ and *S*_max_) are considered.

## Results

### Pure-state GTE distribution

Let us now discuss the relation between *π*_*ABC*_ and *τ* for pure states in details. For this purpose it is convenient to use the equality ${{\mathscr{N}}}_{i(jk)}(\psi )={{\mathscr{C}}}_{i(jk)}(\psi )$ proven in refs^41,61^, where *i*, *j*, *k* = {*A*, *B*, *C*}. Then, *π*_*ABC*_(*ψ*) can be written as follow

8 $${\pi }_{ABC}(\psi )=\tau (\psi )+\frac{2}{3}\,\sum _{i,j}\,({{\mathscr{C}}}_{ij}^{2}(\psi )-{{\mathscr{N}}}_{ij}^{2}(\psi )),$$

where the sum is taken over all pairs *ij*. As we see, the difference between *π*_*ABC*_ and *τ* is related to the simultaneous estimation of entanglement in all biqubit subsystems. It is known^62^, that for two-qubit states the following relations linking the concurrence and negativity are satisfied, $f({{\mathscr{C}}}_{ij})\le {{\mathscr{N}}}_{ij}\le {{\mathscr{C}}}_{ij}$, where $f({{\mathscr{C}}}_{ij})\equiv \sqrt{{(1-{{\mathscr{C}}}_{ij})}^{2}+{{\mathscr{C}}}_{ij}^{2}}-(1-{{\mathscr{C}}}_{ij})$. Consequently, (i) ${\pi }_{ABC}\equiv \tau$ iff one has ${{\mathscr{C}}}_{ij}={{\mathscr{N}}}_{ij}$ for all bipartite subsystems of a tripartite state |*ψ*〉, if such states exist in the entire range of *τ*. Moreover, since the negativity ${{\mathscr{N}}}_{ij}$ can never exceed the concurrence ${{\mathscr{C}}}_{ij}$, the above condition determines the lower bound of the GTE distribution (i.e., *π*_*ABC*_ vs *τ*). (ii) Similarly, one may expect that the upper bound of the GTE distribution is determined by the simultaneous fulfillment of the condition ${{\mathscr{N}}}_{ij}=f({{\mathscr{C}}}_{ij})$ for all bipartite subsystems (if such states exist for any value of *τ*).

In order to verify these assumptions, it is convenient to represent an arbitrary three-qubit pure state |*ψ*〉 in the canonical parametrization proposed by Acín *et al*.^63^.

9 $$|\psi \rangle =d|000\rangle +r{e}^{i\varphi }|100\rangle +a|101\rangle +b|110\rangle +c|111\rangle ,$$

with real *a*, *b*, *c*, *d*, *r* ≥ 0, 0 ≤ *ϕ* ≤ *π* and standard normalization. For such state *τ* and all ${{\mathscr{C}}}_{ij}$ can be easily computed as

10 $$\tau =4{c}^{2}{d}^{2},\,{{\mathscr{C}}}_{AB}=2bd,\,{{\mathscr{C}}}_{AC}=2ad,\,{{\mathscr{C}}}_{BC}=2\sqrt{{a}^{2}{b}^{2}+{c}^{2}{r}^{2}-2abcr\,\cos \,\varphi }.$$

The task of finding the analytical formula of the negativity ${{\mathscr{N}}}_{ij}$ which is defined as twice the sum of the negative eigenvalue of the partially transposed state ${\rho }_{ij}^{T}$, is however not as simple in general case. To derive it we use the fact that for two-qubit state ${\rho }_{ij}^{T}$ has no more than one negative eigenvalue, and thus the negativity satisfies the quartic polynomial equation^48^

11 $${\rm{\det }}({\rho }_{ij}^{T}-{{\mathscr{N}}}_{ij}{I}_{4})=0,$$

where *I*_4_ is 4 × 4 identity matrix. For clarity, let us write this quartic explicitly for *ij* = *AB*

12 $$\begin{array}{l}{{\mathscr{N}}}_{AB}^{4}+2{{\mathscr{N}}}_{AB}^{3}+4[{a}^{2}({b}^{2}+{d}^{2})+{c}^{2}({d}^{2}+{r}^{2})-2abcr\,\cos \,\varphi ]{{\mathscr{N}}}_{AB}^{2}\\ \,-\,8b{d}^{2}[b-2{a}^{2}b+2acr\,\cos \,\varphi ]{{\mathscr{N}}}_{AB}-16{b}^{2}({b}^{2}+{c}^{2}){d}^{4}=0.\end{array}$$

We can now replace ${{\mathscr{N}}}_{ij}$ in Eq. (11) by ${{\mathscr{C}}}_{ij}$ given in Eq. (10), what yields to

13a $${a}^{2}{d}^{2}[{b}^{2}{(a+d)}^{2}+{c}^{2}{r}^{2}-2bc(a+d)r\,\cos \,\varphi ]=0,$$

13b $${b}^{2}{d}^{2}[{a}^{2}{(b+d)}^{2}+{c}^{2}{r}^{2}-2ac(b+d)r\,\cos \,\varphi ]=0,$$

13c $$\begin{array}{l}{d}^{2}[2({a}^{2}{b}^{2}-abcr\,\cos \,\varphi )\sqrt{{a}^{2}{b}^{2}+{c}^{2}{r}^{2}-2abcr\,\cos \,\varphi }\\ \,+\,({a}^{2}+{b}^{2})\,({a}^{2}{b}^{2}+{c}^{2}{r}^{2}-2abcr\,\cos \,\varphi )]=0.\end{array}$$

The existence of the lower bound of the GTE distribution is confirmed if one can find real positive amplitudes *a*, *b*, *c*, *d*, *r* and phase *ϕ* which satisfy Eq. (13a–c) for any value of *τ*.

We note that there are two important examples of states that provide the lower bound, namely the GGHZ and MS states. The GGHZ states are defined as, |*ψ*_*G*_〉 = *d*|000〉 + *c*|111〉. The appropriate entanglement measurements are ${{\mathscr{C}}}_{ij}({\psi }_{G})={{\mathscr{N}}}_{ij}({\psi }_{G})=0$ for all pairs *ij* and the three-tangle is simply given by *τ* = 4*c*^2^*d*^2^ (see Eq. (10)). The MS states, $|{\psi }_{M}\rangle =\frac{1}{\sqrt{2}}|000\rangle +b|110\rangle +c|111\rangle )$, exhibit nonzero ${{\mathscr{C}}}_{AB}({\psi }_{M})={{\mathscr{N}}}_{AB}({\psi }_{M})=\sqrt{2}b$ and *τ*(*ψ*_*M*_) = 2*c*^2^. As we see, since *c* and *d* can take any real positive value (up to the normalization constraints), both the GGHZ and MS states cover the entire available range of *τ*, what confirms the correctness of previous assumption.

Note that both the GGHZ and MS states belong to the three-parameter GHZ subfamily, $$|{\rm{\Phi }}\rangle =\,\cos \,{\theta }_{1}$$$$|(\begin{array}{c}1\\ 0\end{array})(\begin{array}{c}1\\ 0\end{array})(\begin{array}{c}1\\ 0\end{array})\rangle +\,\sin \,{\theta }_{1}\,|(\begin{array}{c}0\\ 1\end{array})(\begin{array}{c}\cos \,{\theta }_{2}\\ \sin \,{\theta }_{2}\end{array})(\begin{array}{c}\cos \,{\theta }_{3}\\ \sin \,{\theta }_{3}\end{array}\rangle $$ discussed in ref.^15^. However, |Φ〉 does not indicate the realization of the lower bound in general what can be easily verify via Eq. (13a–c).

In the case of the upper bound of the GTE distribution, let us first analyze *τ* = 0. From Eq. (10) one can immediately notice that the only promising states for the upper bound are given by *c* = 0 and *d* ≠ 0, i.e. the states that belong to the W-class (GW)^12^. The straightforward calculations provide the fulfillment of Eq. (11) for ${{\mathscr{N}}}_{ij}=f({{\mathscr{C}}}_{ij})$ when *c* = *r* = 0 and $a=b=d=\frac{1}{\sqrt{3}}$. Such state is called the W state^12^. One can also confirm this result by writing the W-class in the standard form |*ψ*_*W*_〉 = *μ*_100_|100〉 + *μ*_010_|010〉 + *μ*_001_|001〉. For this parametrization one has ${{\mathscr{C}}}_{AB}({\psi }_{W})=2|{\mu }_{100}{\mu }_{010}|$, ${{\mathscr{C}}}_{AC}({\psi }_{W})=2|{\mu }_{100}{\mu }_{001}|$ and ${{\mathscr{C}}}_{BC}({\psi }_{W})=2|{\mu }_{010}{\mu }_{001}|$. In the same time, ${{\mathscr{N}}}_{AB}({\psi }_{W})=\sqrt{{\mu }_{001}^{4}+4{\mu }_{100}^{2}{\mu }_{010}^{2}}-{\mu }_{001}^{2}$, standard algebra, that the maximum possible value of *π*_*ABC*_(*ψ*_*W*_) is reached for ${\mu }_{100}={\mu }_{010}={\mu }_{001}=\frac{1}{\sqrt{3}}$, i.e., for the W state. Moreover, for the W state the condition ${{\mathscr{N}}}_{ij}({\psi }_{W})=f({{\mathscr{C}}}_{ij}({\psi }_{W}))$ is satisfied for all pairs *ij* and hence, the W state determines the upper bound on *π*_*ABC*_ for *τ* = 0.

When *τ* > 0 the estimation of the upper bound is much more complicated. Due to the length of the calculations we omit the details of our analysis and present only a general schema. However, the quality of our results are confirmed by numerical calculations presented in this paper. Our calculations are based on the method of Lagrange multipliers with ${\sum }_{i,j}\,({{\mathscr{C}}}_{ij}^{2}(\psi )-{{\mathscr{N}}}_{ij}^{2}(\psi ))$ taken as a target function subject to five constraints:

14 $$\begin{array}{rcl} {\mathcal L}  & = & \sum _{ij}\,({{\mathscr{C}}}_{ij}^{2}(\psi )-{{\mathscr{N}}}_{ij}^{2}(\psi ))+{\lambda }_{1}{g}_{AB}+{\lambda }_{2}{g}_{AC}+{\lambda }_{3}{g}_{AB}\\  &  & +\,{\lambda }_{4}(4{c}^{2}{d}^{2}-\tau )+{\lambda }_{5}({a}^{2}+{b}^{2}+{c}^{2}+{d}^{2}+{r}^{2}-1),\end{array}$$

where ${g}_{ij}={\rm{\det }}({\rho }_{ij}^{T}-{{\mathscr{N}}}_{ij}{I}_{4})$, ${{\mathscr{C}}}_{ij}$ are given by Eq. (10) and *τ* is considered as a constant value. Based on the previous discussion one can also assume *c*, *d* ≠ 0.

Differentiating ${\mathcal L}$ with respect to *x* = {*a*, *b*, *c*, *d*, *r*, *ϕ*} and setting $\partial {{\mathscr{N}}}_{ij}/\partial x=0$ gives six polynomial equations (sufficient conditions). Analyzing the stationary points of the partial derivatives of Lagrangian ${\mathcal L}$ we have determined the upper bound states |*ψ*_*T*_〉 that are described by the amplitudes

15 $$\begin{array}{rcl}a & = & b=\frac{1-2{d}^{2}+\sqrt{1+4{d}^{2}-12{d}^{4}}}{4d},\,c=a\sqrt{\frac{6{d}^{2}-1-\sqrt{1+4{d}^{2}-12{d}^{4}}}{2-4{d}^{2}}},\\ r & = & \frac{1-2{d}^{2}}{2d}\sqrt{\frac{6{d}^{2}-1-\sqrt{1+4{d}^{2}-12{d}^{4}}}{2-4{d}^{2}}}\end{array}$$

and phase *ϕ* = *π*, where $1/\sqrt{3}\le d\le 1/\sqrt{2}$. For $d=1/\sqrt{2}$ Eq. (15) lead to the GHZ state i.e. *a* = *b* = *r* = 0 and $c=1/\sqrt{2}$, if the left-handed limit with respect to *d* is taken. Note that the biggest disadvantage of |*ψ*_*T*_〉 presented above is undoubtedly the complicated form which causes further difficulties in potential application of |*ψ*_*T*_〉 in quantum information protocols. To overcome this problem, we define $d=\sqrt{{a}_{T}({a}_{T}+\sqrt{1-3{a}_{T}^{2}})}$ and apply the inverse transformation to the one given by Acín *et al*. in ref.^63^. Then, the states |*ψ*_*T*_〉 can be written in a standard form as

16 $$|{\psi }_{T}\rangle ={a}_{T}(|110\rangle +|101\rangle +|011\rangle )+{b}_{T}|000\rangle ,$$

where ${b}_{T}=\sqrt{1-3{a}_{T}^{2}}$ and $1/2\le {a}_{T}\le 1/\sqrt{3}$. These states are known as tetrahedral states (or generalized triple states)^64^ and can be interpreted as a proper superposition of the reverse W state and the vacuum state. For the tetrahedral states we have found ${{\mathscr{C}}}_{ij}({\psi }_{T})={{\mathscr{C}}}_{T}=2{a}_{T}({a}_{T}-{b}_{T})$ and ${{\mathscr{N}}}_{ij}({\psi }_{T})={{\mathscr{N}}}_{T}=-\,1+2{a}_{T}^{2}+\sqrt{1-8{a}_{T}^{2}+20{a}_{T}^{4}}$ for all *ij*. Using these expressions it is easy to show that ${{\mathscr{N}}}_{ij}({\psi }_{T})\ne f({{\mathscr{C}}}_{ij}({\psi }_{T}))$, except ${a}_{T}=1/\sqrt{3}$ (i.e. *τ* = 0). In other words, for *τ* > 0 there is no three-qubit pure states with rank-2 quasi-distillable reduced states *ρ*_*ij*_^62^. Finally, both GTE monotonies are given as

17 $$\tau ({\psi }_{T})=16{a}_{T}^{3}{b}_{T},$$

18 $${\pi }_{ABC}({\psi }_{T})=\tau ({\psi }_{T})+2({{\mathscr{C}}}_{T}^{2}-{{\mathscr{N}}}_{T}^{2}).$$

It should be emphasized that the tetrahedral states have been previously recognized as a lower bound of the primary yield of GHZ states from the infinitesimal distillation protocol in terms of *τ* while the GGHZ and MS states stands for the upper bound^64,65^.

**Figure 1:**
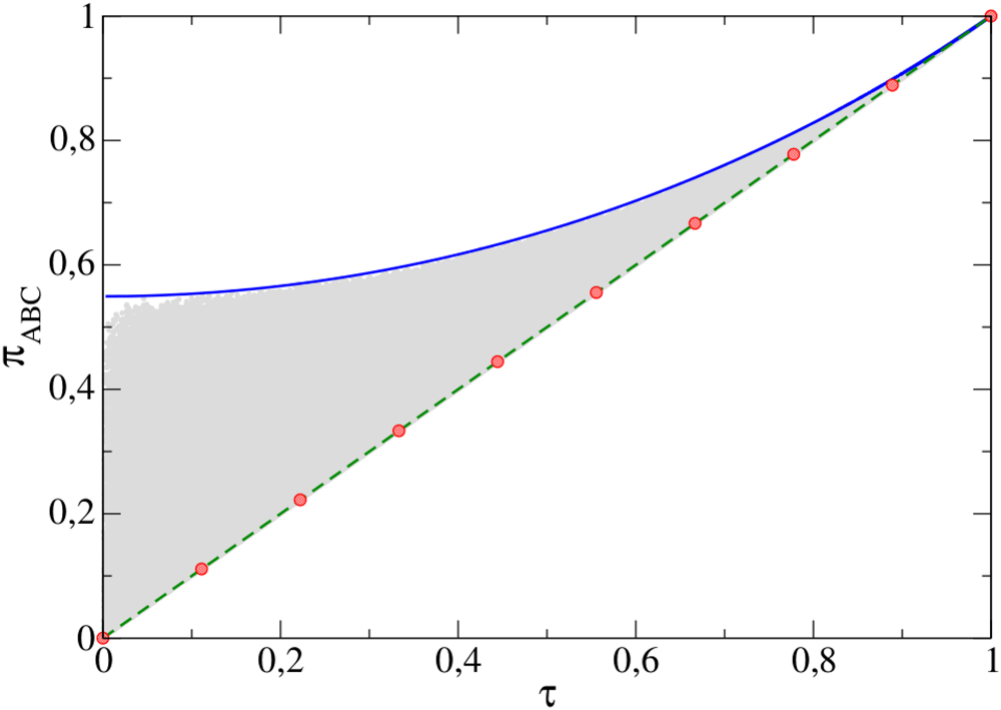
Range of values of *π*_*ABC*_ for a given *τ*. The dashed line represents the theoretical values determined for the GGHZ state wheres circle symbols indicate the MS states. The solid curve depicts the values of |*ψ*_*T*_〉 (Eqs (17) and (18)). Gray area corresponds to all admissible values achieved for 10^7^ randomly generated three-qubit states.

In Fig. 1 the GTE distribution for the previously discussed group of states is presented. The gray area in the figure shows all possible values of *π*_*ABC*_ and *τ* for 10^7^ randomly generated tripartite pure states performed to check all of our predictions.

### Restriction on the nonlocal correlations

Now, we are in the position to present the main result of this paper. As it was shown above, the lower bound of the GTE distribution can be formed by the GGHZ and MS states. In the first case one can find that the Svetlichny inequality writes ${S}_{{\rm{\max }}}({\psi }_{G})=8\sqrt{2}cd$ for *c*^2^*d*^2^ ≥ 1/12 and ${S}_{{\rm{\max }}}({\psi }_{G})=4\sqrt{1-4{c}^{2}{d}^{2}}$ for all other cases, while for the MS states we have ${S}_{{\rm{\max }}}({\psi }_{M})=4\sqrt{1+2{c}^{2}}$^17^. On the other hand, *T*_max_(*ψ*_*G*_) = 4*cd* when *cd* ≥ 1/4 and *T*_max_(*ψ*_*G*_) = 1 for all other cases provided *cd* ≤ 1/4, which is nothing more than $\sqrt{{T}_{{\rm{\max }}}^{^{\prime} }({\psi }_{G})}$ described in ref.^59^. The MS states give ${T}_{{\rm{\max }}}({\psi }_{M})=\sqrt{{T}_{{\rm{\max }}}^{^{\prime} }({\psi }_{M})}=\sqrt{2(1+2{c}^{2})}$. The last equality (similarly as for GGHZ states) arises from the fact that the two vectors in Eq. (6), i.e. $({g}_{x}^{A}{g}_{x}^{B}{g}_{x}^{C},\ldots ,{g}_{y}^{A}{g}_{y}^{B}{g}_{y}^{C})$ and (|*T*_*xxx*_|, …, |*T*_*yyy*_|) can be made parallel and hence, inequality (7) turns out to be both necessary and sufficient condition for an arbitrary three-qubit state to satisfy the general Bell inequality discussed in ref.^3^. When considering correlation tensor $\hat{T}$ in local coordinates systems expressed as a sequences of rotation around $\overrightarrow{z}-\overrightarrow{x}^{\prime} -\overrightarrow{z}^{\prime\prime}$ axes (defined by three local angles {*φ*_*i*_,*ϑ*_*i*_,*γ*_*i*_}), such two parallel vectors are given by {*φ*_1_, *ϑ*_1_, *γ*_1_} = {0, 0, 0}, $\{{\phi }_{1},\,{\vartheta }_{1},\,{\gamma }_{1}\}=\{0,\,0,\,\tfrac{\pi }{4}\}$, $\{{\phi }_{3},\,{\vartheta }_{3},\,{\gamma }_{3}\}=\{\tfrac{\pi }{2},\,\arccos (\sqrt{2}c,\,\tfrac{\pi }{2}\}$ and three vectors ${\overrightarrow{g}}^{1}=(\frac{\sqrt{2}c}{\sqrt{1+2{c}^{2}}},\,\frac{\sqrt{2}c}{\sqrt{1+2{c}^{2}}})$, ${\overrightarrow{g}}^{2}={\overrightarrow{g}}^{3}=(\frac{1}{\sqrt{2}},\,\frac{1}{\sqrt{2}})$.

**Figure 2:**
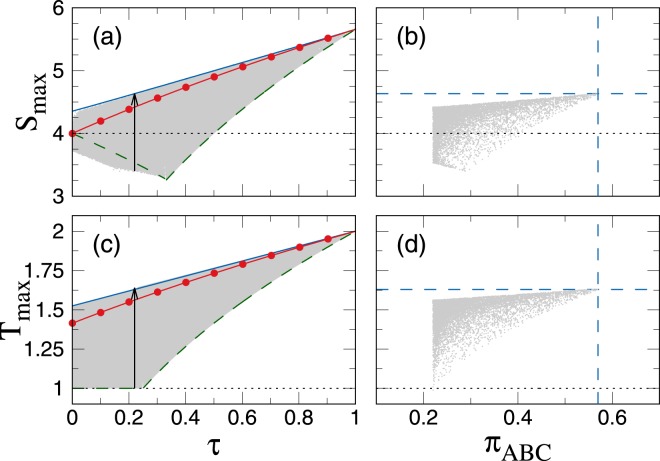
The maximum violation of Svetlichny (*S*_max_) and generalized Bell (*T*_max_) inequalities for a given *τ* (panels (a) and (c)). Dashed lines represent the theoretical values determined for the GGHZ state whereas line with circle symbols indicate the MS states. Solid lines depicts the values for the |*ψ*_*T*_〉 states (given by Eqs (19) and (20), respectively). Gray areas correspond to all admissible values achieved for 10^7^ random three-qubit states and dotted lines indicate the local variable theories limits. Black vectors denote cross sections of *S*_max_ and *T*_max_ for *τ* = 0.22 presented in panels (b) and (d), respectively. In these panels, gray points represent all admissible values of *S*_max_ and *T*_max_ achieved for randomly generated three-qubit states {|*ψ*_*n*_〉} which correspond to *τ* = 0.22. The intersection of dashed lines stands for the analytical results of the |*ψ*_*T*_〉_*τ*=0.22_ state.

As we can see in Fig. 2(a,c), *S*_max_ (*T*_max_) vs *τ* for the GGHZ and MS states does not overlap what is in contrast to the GTE distribution. Here, these two functions specify the area of achievable values of *S*_max_(Φ) and *T*_max_(Φ) for a given amount of *τ* and the GGHZ and MS states represent the boundary states. For instance, the following relation for the Svetlichny inequality has been established by Emary *et al*.^15^, $|\tfrac{1}{16}{S}_{{\rm{\max }}}^{2}({\rm{\Phi }})-1|\le \tau \le \tfrac{1}{32}{S}_{{\rm{\max }}}^{2}({\rm{\Phi }})$ (proven analytically in ref.^17^). However, as we see in Fig. 2 (the gray areas) there exist pure states that violate *T*_max_ and *S*_max_ stronger than the MS states do. To confirm this fact the W-class written in the standard form has been examined. Based on the analytical formula of *S*_max_(*ψ*_*W*_) derived in ref.^66^, one can find that the maximum expectation value of *S*_max_(*ψ*_*W*_) is reached for the W state, *S*_max_(*W*) ≈ 4.35, what is greater than the non-locality of MS states, ${{S}_{{\rm{\max }}}({\psi }_{M})|}_{\tau =0}=4$. Similar calculations can be done for *T*_max_ given by Eq. (6) and it can be shown that ${\rm{\max }}\,\{{T}_{{\rm{\max }}}({\psi }_{W})\}={T}_{{\rm{\max }}}(W)\approx 1.523$ whereas ${{T}_{{\rm{\max }}}({\psi }_{M})|}_{\tau =0}=\sqrt{2}$. Note that in contrast to previous states *T*_max_(*W*) is smaller than $\sqrt{{T}_{{\rm{\max }}}^{^{\prime} }(W)}=\sqrt{\frac{7}{3}}$ presented in ref.^60^.

Since the W state determines the upper bound of GTE distribution for *τ* = 0, one can ask whether the three-*π* can be used in order to determine the Tsirelson-like bound for all *τ*. To answer this question let us examine states described by Eq. (9) where parameters *c* and *d* are chosen in such a way to provide a given value of *τ*. Due to the large number of free parameters that should be optimized, we restrict our research to numerical analysis. For this purpose, a set of random states {|*ψ*_*n*_〉} corresponding to constant *τ* is generated. It is natural that these states correspond to different values of *π*_*ABC*_ and hence, one can sort *S*_max_({|*ψ*_*n*_〉}) and *T*_max_({|*ψ*_*n*_〉}) in ascending order with respect to *π*_*ABC*_. Such results represent a cross section of *S*_max_ vs *τ* (*T*_max_ vs *τ*). The exemplary outcomes have been presented in Fig. 2(b,d). As we see, when *π*_*ABC*_ increases the interval width of *S*_max_({|*ψ*_*n*_〉}) and *T*_max_({|*ψ*_*n*_〉}) for a given *π*_*ABC*_ decreases. However, the upper bound of such interval always rises. Consequently, when *π*_*ABC*_({|*ψ*_*n*_〉}) approaches its maximum value the Tsirelson-like bound of *S*_max_ and *T*_max_ for a given amount of *τ* is reached. These results give a strong evidence that the upper bound states |*ψ*_*T*_〉 yield the maximum possible value of *S*_max_ and *T*_max_ for a given amount of *τ*. For the |*ψ*_*T*_〉 states we have found that both nonlocality measures (Eqs (5) and (6)) are given as

19 $${S}_{{\rm{\max }}}({\psi }_{T})=\frac{16\sqrt{2}{[{a}_{T}({a}_{T}+{b}_{T})]}^{3/2}}{\sqrt{6{a}_{T}({a}_{T}+{b}_{T})-1}},$$

and

20 $$\begin{array}{rcl}{T}_{{\rm{\max }}}({\psi }_{T}) & = & \mathop{{\rm{\max }}}\limits_{{g}_{x},\gamma }\,(\tfrac{1}{4}\{3[1-2{a}_{T}({a}_{T}+{b}_{T})]\,({g}_{x}+{g}_{y})\,[{g}_{x}({g}_{x}-{g}_{y})\,\cos \,\gamma +{g}_{y}({g}_{y}-{g}_{x})\,\sin \,\gamma ]\\  &  & -\,[1+6{a}_{T}({a}_{T}+{b}_{T})]\,[{g}_{x}(1+2{g}_{y}^{2})\,\cos (3\gamma )+{g}_{y}(2{g}_{x}^{2}+1)\,\sin (3\gamma )]\}),\end{array}$$

where ${b}_{T}=\sqrt{1-3{a}_{T}^{2}}$, $1/2\le {a}_{T}\le 1/\sqrt{3}$ and *g*_*x*_, *g*_*y*_ are the components of the unit vector. What is important, all variables that enter Eq. (20) (namely, *g*_*x*_, *g*_*y*_ and *γ*) are functions of *a*_*T*_. For that reason, the final form of *T*_max_(*ψ*_*T*_) is far from being easily presented. However, for given value of *a*_*T*_ Eq. (20) can be maximized within straightforward calculations. In particular, one can simplify the problem and assume $\gamma =\frac{5\pi }{12}$ which ensures underestimation of *T*_max_(*ψ*_*T*_) of less than 10^−5^. On the other hand, the upper bound of *T*_max_(*ψ*_*T*_) is given by $\sqrt{{T}_{{\rm{\max }}}^{^{\prime} }({\psi }_{T})}$, where

21 $${T}_{{\rm{\max }}}^{^{\prime} }({\psi }_{T})=\sqrt{12{a}_{T}^{2}(1-2{a}_{T}({a}_{T}-{b}_{T}))+1}$$

which overestimate Eq. (20) not more than 10^−3^ when ${a}_{T}=1/\sqrt{3}$ and yield an exact value for *a*_*T*_ = 1/2. It is worth mentioning that although ${T}_{{\rm{\max }}}^{^{\prime} }$ is just a sufficient constraints for a state to have a local correlation function, ${T}_{{\rm{\max }}}^{^{\prime} }({\psi }_{T})$ exhibits similar behavior as *T*_max_(*ψ*_*T*_). i.e. ${T}_{{\rm{\max }}}^{^{\prime} }(\{|{\psi }_{n}\rangle \})$ sorted in ascending order with respect to *π*_*ABC*_ yield ${T}_{{\rm{\max }}}^{^{\prime} }({\psi }_{T})$ for a given value *τ*.

As we can see in Fig. 2, our results are in perfect agreement with numerical calculations for both *S*_max_(*ψ*_*T*_) and *T*_max_(*ψ*_*T*_) in the entire range of *τ*. Therefore, even if states |*ψ*_*T*_〉 do not have the status of the exact analytical boundary, indisputable closeness to the upper limit of *S*_max_(*ψ*_*T*_) and *T*_max_(*ψ*_*T*_) allows one to consider |*ψ*_*T*_〉 as the Tsirelson-like bounds. Furthermore, the above observation provides an interesting relations between three concepts i.e. tripartite entanglement monotony, GHZ destilation and non-locality what makes those states promising for further studies in specific information processing tasks. In particular, the tetrahedral states have been recently successfully used for perfect controlled teleportation of equatorial states^39^.

Finally, it is important to mention that the numerical calculations described above reveal another interesting feature i.e. for all analyzed states the quantity $\sqrt{{T}_{{\rm{\max }}}^{^{\prime} }}-{T}_{{\rm{\max }}}\lesssim {10}^{-2}$. Consequently, ${T}_{{\rm{\max }}}^{^{\prime} }\lesssim {({T}_{{\rm{\max }}}+{10}^{-2})}^{2}$ and since *T*_max_ > 1 implies the existance of quantum correlation than, based on Eq. (7) one can propose a sufficient condition of nonlocality

22 $$\sum _{\alpha ,\beta ,\gamma }\,{T}_{\alpha \beta \gamma }^{2} > 1.01,$$

which is simplest than Eq. (6). However, further studies in this field are needed.

### GHZ-symmetric mixed states

Let us now verify whether the conciliation between the restriction of non-locality and *π*_*ABC*_ can be observed for mixed states. For that reason we consider an example of the mixed state, namely the GHZ-symmetric states, i.e. the family of all mixed states *ρ*^*S*^ that obey the same symmetry as the GHZ states^56,57^. In particular, this family include the three-qubit generalized Werner states. The GHZ-symmetric state are fully specified by two independent real parameters (*x*, *y*). The set of states *ρ*^*S*^(*x*, *y*) forms a triangle in the (*x*, *y*) plane limited by −$$\frac{1}{4\sqrt{3}}\le y\le \frac{\sqrt{3}}{4}$$ and $$|x|\le \frac{1}{8}+\frac{\sqrt{3}}{2}y$$. The three-tangle *τ*(*x*, *y*) = 0 for *x* ≤ *x*^*W*^, *y* ≤ *y*^*W*^ where $${x}^{W}=\frac{{v}^{5}+8{v}^{3}}{8(4-{v}^{2})}$$, $${y}^{W}=\frac{\sqrt{3}}{4}\frac{4-{v}^{2}-{v}^{4}}{4-{v}^{2}}$$ and −1 ≤ *v* ≤ 1^57^. For *x* > *x*^*W*^ and *y* > *y*^*W*^ all states *ρ*^*S*^(*x*, *y*) which are related with a given amount of *τ* lie on the parametrized line $$\{{x}_{\tau },{y}_{\tau }\}=\{(1/2-{x}^{W})\tau +{x}^{W},(\sqrt{3}/4-{y}^{W})\tau +{y}^{W}\}$$. Furthermore, all other quantities are given by $${\pi }_{ABC}(x,y)=\,{\rm{\max }}\,\{0,-\,\tfrac{1}{8}+|x|+\tfrac{1}{2\sqrt{3}}y\}$$ and $${T}_{{\rm{\max }}}(x,y)=\sqrt{{T^{\prime} }_{{\rm{\max }}}(x,y)}=4|x|$$, $${S}_{{\rm{\max }}}(x,y)=$$$$8\sqrt{2}|x|$$^67^. Due to the symmetry of GHZ states one can assume, without loss of generality, that *x* ≥ 0. As we see, *π*_*ABC*_ is a linear-dependent function of *x* and *y*. Consequently, the maximal value of *π*_*ABC*_(*x*_*τ*_, *y*_*τ*_) for *τ* = 0 is located at $$({x}_{0},{y}_{0})=(3/8,\sqrt{3}/6)$$ (what corresponds to *v* = 1). This is also the upper limit of *T*_max_(*x*_*τ*_, *y*_*τ*_) and *S*_max_(*x*_*τ*_, *y*_*τ*_) since for a given *y*_0_ one cannot increase *x* due to the limitation of the (*x*, *y*) plane described above and any changes of *y*_0_ (restricted to *τ* = 0) causes decrease of *x*. Analogously, one can show that for *τ* > 0 the upper border of *π*_*ABC*_(*x*_*τ*_, *y*_*τ*_) is given by *x*_*τ*_ = (3 + *τ*)/8 and $${y}_{\tau }=(2+\tau )/(4\sqrt{3})$$. Also in this case it determines the maximal values of *T*_max_(*x*_*τ*_, *y*_*τ*_) and *S*_max_(*x*_*τ*_, *y*_*τ*_). In other words, the upper border of *π*_*ABC*_(*x*, *y*) vs *τ*(*x*, *y*) determines the restriction on the nonlocal correlations for all *τ* and the GHZ-symmetric states share this property with the pure states.

## Conclusions

In summary, we have solved several problems about the GTE measures and their relations with non-locality. First of all, we have established the restrictions of violations of Bell-type inequalities for tripartite states with a given entanglement properties (quantified by the three-tangle). We have derived a one-parameter family of states that yield the extremal quantum values of the Bell-type inequalities. Those states are known as tetrahedral states and reveal extremal properties also with respect to the GHZ-states distillation and the GTE distribution, i.e., the highest difference in the estimation of GTE by mean of three-tangle and three-*π*. As a consequence of the last one, the Tsirelson-like boundary can be found by simultaneous use of *π*_*ABC*_ and *τ*. This observation has also been verify for the GHZ-symmetric mixed states and we have found a good agreement with our predictions. Therefore, the proposed method allows to characterize the limits of non-locality which are quantum mechanically possible for a state with given entanglement properties with help of a computable measure, *π*_*ABC*_. We note that further studies of the relation between GTE distribution and the Tsirelson-like boundary for the mixed states are complicated by the need of the exact results of the mixed-state three-tangle. Nonetheless, we believe that our results shed a new light on the understanding of both GTE monotonous discussed in this paper and their connection with non-locality. Especially, the tetrahedral states seem to have important meanings for further studies in the field of quantum theory. Furthermore, the analysis presented in this paper seems to be promising in further extension to the N-partite case.

## Methods

### Derivation of *S*_max_(*ψ*_*T*_) and *T*_max_(*ψ*_*T*_)

In order to prove Eq. (19), let $\overrightarrow{a}=(\sin \,{\theta }_{a}\,\cos \,{\varphi }_{a},\,\sin \,{\theta }_{a}\,\sin \,{\varphi }_{a},\,\cos \,{\theta }_{a})$, and likewise for similarly defined terms. Furthermore, by defining unitary vectors $\overrightarrow{k}$ and $\overrightarrow{k}\,^{\prime}$ such that $2\overrightarrow{k}\,\cos \,t=\overrightarrow{b}+\overrightarrow{b^{\prime} }$ and $2\overrightarrow{k^{\prime} }\,\sin \,t=\overrightarrow{b}-\overrightarrow{b^{\prime} }$, one can simplify Eq. (5) for |*ψ*_*T*_〉 as

23 $$\begin{array}{rcl}S({\psi }_{T}) & = & 2|\,\cos \,t[\langle {V}_{A}{V}_{K}{V}_{C}\rangle -\langle {V}_{A}{V}_{K}^{^{\prime} }{V}_{C}^{^{\prime} }\rangle ]+\,\sin \,t[\langle {V}_{A}{V}_{K}^{^{\prime} }{V}_{C}^{^{\prime} }\rangle -\langle {V}_{A}^{^{\prime} }{V}_{K}^{^{\prime} }{V}_{C}\rangle ]|\\  & \le  & 2|{\{{[\langle {V}_{A}{V}_{K}{V}_{C}\rangle -\langle {V}_{A}{V}_{K}^{^{\prime} }{V}_{C}^{^{\prime} }\rangle ]}^{2}+{[\langle {V}_{A}{V}_{K}^{^{\prime} }{V}_{C}^{^{\prime} }\rangle -\langle {V}_{A}^{^{\prime} }{V}_{K}^{^{\prime} }{V}_{C}\rangle ]}^{2}\}}^{1/2}|,\end{array}$$

where ${V}_{K}=\overrightarrow{k}\cdot {\overrightarrow{\sigma }}_{B}$, ${V}_{K}^{^{\prime} }=\overrightarrow{k}^{\prime} \cdot {\overrightarrow{\sigma }}_{B}$ and we have used the Cauchy inequality, *x* cos *t* + *y* sin *t* ≤ (*x*^2^ + *y*^2^)^1/2^ (see, ref.^17^). Note that by definition $\overrightarrow{k}\cdot \overrightarrow{k^{\prime} }=\,\cos \,{\theta }_{k}\,\cos \,{\theta }_{k^{\prime} }+\,\sin \,{\theta }_{k}\,\sin \,{\theta }_{k^{\prime} }\,\cos ({\varphi }_{k}-{\varphi }_{k^{\prime} })=0$.

The first term in Eq. (23) with respect to |*ψ*_*T*_〉 gives

24 $$\begin{array}{rcl}\langle {V}_{A}{V}_{K}{V}_{C}\rangle  & = & 2{a}_{T}\,\sin \,{\theta }_{a}[{{\rm{\Omega }}}_{ak}\,\cos \,{\theta }_{c}\,\sin \,{\theta }_{k}+{{\rm{\Omega }}}_{ac}\,\cos \,{\theta }_{k}\,\sin \,{\theta }_{c}]\\  &  & +\,\cos \,{\theta }_{a}[2{a}_{T}{{\rm{\Omega }}}_{kc}\,\sin \,{\theta }_{k}\,\sin \,{\theta }_{c}-\,\cos \,{\theta }_{k}\,\cos \,{\theta }_{c}],\end{array}$$

where Ω_*ij*_ = *a*_*T*_ cos(*ϕ*_*i*_ − *ϕ*_*j*_) − *b*_*T*_ cos(*ϕ*_*i*_ + *ϕ*_*j*_). What is important, other terms in Eq. (23) has a similar form as 〈*V*_*A*_*V*_*K*_*V*_*C*_〉. Therefore, the inherent symmetry in Eq. (24) implies that *S*_max_(*ψ*_*T*_) is obtained when *ϕ*_*i*_ − *ϕ*_*j*_ = 0 and *ϕ*_*i*_ + *ϕ*_*j*_ = *π* i.e. when all ${\varphi }_{i}={\varphi }_{i^{\prime} }=\frac{\pi }{2}$. Moreover, this outcome entails that the constraint $\overrightarrow{k}\cdot \overrightarrow{k^{\prime} }=0$ is fulfilled when ${\theta }_{k}={\theta }_{k^{\prime} }+\frac{\pi }{2}$. Inserting all these result to Eq. (23) and putting ${\theta }_{k}=\frac{\pi }{2}$ we have

25 $$\begin{array}{rcl}S({\psi }_{T}) & \le  & 2|\{[\cos \,{\theta }_{a^{\prime} }\,\cos \,{\theta }_{c}+\,\cos \,{\theta }_{a}\,\cos \,{\theta }_{c^{\prime} }-2{a}_{T}({a}_{T}+{b}_{T})\\  &  & \times \,(\sin \,{\theta }_{a^{\prime} }\,\sin \,{\theta }_{c}+\,\sin \,{\theta }_{a}\,\sin \,{\theta }_{c^{\prime} }){]}^{2}+4{a}_{T}^{2}{({a}_{T}+{b}_{T})}^{2}\\  &  & \times \,{[\sin ({\theta }_{a}+{\theta }_{c})-\sin ({\theta }_{a^{\prime} }+{\theta }_{c^{\prime} })]}^{2}{\}}^{1/2}|.\end{array}$$

The global maximum of the right-hand side of Eq. (25) occurs when sign(sin *θ*_i_) = −sign(sin *θ*_i′_). Furthermore, due to the fact that all angles are independent variables and by symmetry of Eq. (25) one can put *θ*_*a*_ = *θ*_*c*_ = −*θ*_*a*′_ = −*θ*_*c*′_ = *θ* in order to maximize this expression. As a result one has $S({\psi }_{T})$ ≤ $4|\{{[{\cos }^{2}\theta +2{a}_{T}({a}_{T}+{b}_{T}){\sin }^{2}\theta ]}^{2}$ + $4{a}_{T}^{2}{({a}_{T}+{b}_{T})}^{2}\,\sin (2\theta ){]}^{2}{\}}^{1/2}$ what can be further analyzed by standard algebra. Consequently, when $\theta =-\,\arctan \,(\sqrt{\frac{4{a}_{T}({a}_{T}+{b}_{T})-1}{2{a}_{T}({a}_{t}+{b}_{T})}})$ one reached ${S}_{{\rm{\max }}}({\psi }_{T})\le \frac{16\sqrt{2}{[{a}_{T}({a}_{T}+{b}_{T})]}^{3/2}}{\sqrt{6{a}_{T}({a}_{T}+{b}_{T})-1}}$. We note that other ${\theta }_{k}\ne n\frac{\pi }{2}$, where *n* is an integer number, yields a lower values of *S*(*ψ*_*T*_), so we have established the global maximum of *S*(*ψ*_*T*_). Finally, the set of measurement angles that provides the equality in the above expression is given by ${\varphi }_{m}={\varphi }_{m^{\prime} }=\frac{\pi }{2}$ and ${\theta }_{m}=\pi -{\theta }_{m^{\prime} }=\frac{1}{2}\,\arccos \,(\frac{1-2{a}_{T}({a}_{T}+{b}_{T})}{-1+6{a}_{T}({a}_{T}+{b}_{T})})$ (where *m* = *a*, *b*, *c*) what ends the proof.

Similar analysis can be used to derive Eq. (20). First, the correlation tensor $\hat{T}$ of |*ψ*_*T*_〉 is moved into an arbitrary coordinate system by mean of three Euler rotations, where we use the $\overrightarrow{z}-\overrightarrow{x}^{\prime} -\overrightarrow{z}^{\prime\prime}$ sequences of rotation axes. This allows to introduce three local angles {*φ*_*i*_, *ϑ*_*i*_, *γ*_*i*_} related with the successive Euler rotations. Then, after some simplification one can find that all angles *φ*_*i*_ enter the new correlation tensor $\hat{T}^{\prime\prime}$ in a way given by Ω_*ij*_, so we can put ${\phi }_{i}=\frac{\pi }{2}$. Furthermore, one can easily check that in such case, the elements of $\hat{T}^{\prime\prime}$ can be maximized (up to the absolute value) when all ${\vartheta }_{i}=n\frac{\pi }{2}$. This operation also provides a maximization of *T*_max_(*ψ*_*T*_) since it is a linear function of the absolute values of the elements of $\hat{T}^{\prime\prime}$ (see Eq. (6)). Finally, by the symmetry the maximum of the resulting *T*_max_(*ψ*_*T*_) is reached when all *γ*_*i*_ = *γ* and ${\overrightarrow{g}}^{i}=({g}_{x},{g}_{y})$ what leads to Eq. (20).

## References

[1] Bell JS (1964). On the eistein podolsky rosen paradox. Phys..

[2] Clauser JF, Horne MA, Shimony A, Holt RA (1969). Proposed experiment to test local hidden-variable theories. Phys. Rev. Lett..

[3] Żukowski M, Brukner Č (2002). Bell’s theorem for general n-qubit states. Phys. Rev. Lett..

[4] Svetlichny G (1987). Distinguishing three-body from two-body nonseparability by a bell-type inequality. Phys. Rev. D.

[5] Vedral V (2002). The role of relative entropy in quantum information theory. Rev. Mod. Phys..

[6] Horodecki R, Horodecki P, Horodecki M, Horodecki K (2009). Quantum entanglement. Rev. Mod. Phys..

[7] Werner R (1989). Quantum states with einstein-podolsky-rosen correlations admitting a hidden-variable model. Phys. Rev. A.

[8] Augusiak R, Demianowicz M, Tura J, Acín A (2015). Entanglement and nonlocality are inequivalent for any number of parties. Phys. Rev. Lett..

[9] Gisin N (1991). Bell’s inequality holds for all non-product states. Phys. Lett. A.

[10] Verstraete F, Wolf MM (2002). Entanglement versus bell violations and their behavior under local filtering operations. Phys. Rev. Lett..

[11] Datta C, Agrawal P, Choudhary SK (2017). Measuring higher-dimensional entanglement. Phys. Rev. A.

[12] Dür W, Vidal G, Cirac JI (2000). Three qubits can be entangled in two inequivalent ways. Phys. Rev. A.

[13] Acín A, Bruss D, Lewenstein M, Sanpera A (2001). Classification of mixed three-qubit states. Phys. Rev. Lett..

[14] Tsirelson BS (1987). Quantum analogues of the bell inequalities. the case of two spatially separated domains. J. Sov. Math..

[15] Emary C, Beenakker CWJ (2004). Relation between entanglement measures and bell inequalities for three qubits. Phys. Rev. A.

[16] Navascues M, Pironio S, Acín A (2008). A convergent hierarchy of semidefinite programs characterizing the set of quantum correlations. New J. Phys..

[17] Ghose S, Sinclair N, Debnath S, Rungta P, Stock R (2009). Tripartite entanglement versus tripartite nonlocality in three-qubit greenberger-horne-zeilinger-class states. Phys. Rev. Lett..

[18] Junge M, Palazuelos C (2011). Large violation of bell inequalities with low entanglement. Commun. Math. Phys..

[19] Palazuelos C (2014). On the largest bell violation attainable by a quantum state. J. Funct. Anal..

[20] Horodecki K, Murta G (2015). Bounds on quantum nonlocality via partial transposition. Phys. Rev. A.

[21] Coffman V, Kundu J, Wootters WK (2000). Distributed entanglement. Phys. Rev. A.

[22] Carteret H, Sudbery A (2000). Local symmetry properties of pure three-qubit states. J. Phys. A.

[23] Mermin ND (1990). Extreme quantum entanglement in a superposition of macroscopically distinct states. Phys. Rev. Lett..

[24] Szalay S (2015). Multipartite entanglement measues. Phys. Rev. A.

[25] Huber M, Perarnau-Llobet M, de Vicente JI (2013). Entropy vector formalism and the structure of multidimensional entanglement in multipartite systems. Phys. Rev. A.

[26] Spee C, de Vicente JI, Kraus B (2016). The maximally entangled set of 4-qubit states. J. Math. Phys..

[27] Sauerwein D, Schwaiger K, Cuquet M, de Vicente JI, Kraus B (2015). Source and accessible entanglement of few-body systems. Phys. Rev. A.

[28] Eltschka C, Siewert J (2015). Monogamy equalities for qubit entanglement from lorentz invariance. Phys. Rev. Lett..

[29] Pawlowski M (2010). Security proof for cryptographic protocols based only on the monogamy of bell’s inequality violations. Phys. Rev. A.

[30] Seevinck M (2010). Monogamy of correlations versus monogamy of entanglement. Quantum Inf. Process..

[31] Camalet S (2017). Monogamy inequality for entanglement and local contextuality. Phys. Rev. A..

[32] Camalet S (2017). Monogamy inequality for any local quantum resource and entanglement. Phys. Rev. Lett..

[33] Ferraro A, García-Sáez A, Acín A (2007). Monogamy and ground-state entanglement in highly connected systems. Phys. Rev. A.

[34] Ma X, Dakic B, Naylor W, Zeilinger A, Walther P (2011). Quantum simulation of the wavefunction to probe frustrated heisenberg spin systems. Nat. Phys..

[35] García-Sáez A, Latorre JI (2013). Renormalization group contraction of tensor networks in three dimensions. Phys. Rev. B.

[36] Verlinde E, Verlinde H (2013). Black hole entanglement and quantum error correction. J. High Energy Phys..

[37] Lloyd, S. & Preskill, J. Unitarity of black hole evaporation in final-state projection models. *J*. *High Energy Phys*. **126** (2014).

[38] Eltschka C, Siewert J (2014). Quantifying entanglement resources. J. Phys. A: Math. Theor..

[39] Li X-H, Ghose S (2014). Control power in perfect controlled teleportation via partially entangled channels. Phys. Rev. A.

[40] Jeong K, Kim J, Lee S (2016). Minimal control power of the controlled teleportation. Phys. Rev. A.

[41] Ou Y-C, Fan H (2007). Monogamy inequality in terms of negativity for three-qubit states. Phys. Rev. A.

[42] Horodecki M, Horodecki P, Horodecki R (1996). Separability of mixed states: necessary and sufficient conditions. Phys. Lett. A.

[43] Źyczkowski K, Horodecki P, Sanpera A, Lewenstein M (1998). Volume of the set of separable states. Phys. Rev. A.

[44] Vidal G, Werner RF (2002). Computable measure of entanglement. Phys. Rev. A.

[45] Choi JH, Kim JS (2015). Negativity and strong monogamy of multiparty quantum entanglement beyond qubits. Phys. Rev. A.

[46] Karmakar S, Sen A, Bhar A, Sarkar D (2016). Strong monogamy conjecture in a four-qubit system. Phys. Rev. A.

[47] Kim JS (2016). Strong monogamy of multiparty quantum entanglement for partially coherently superposed states. Phys. Rev. A.

[48] Allen GW, Meyer DA (2017). Polynomial monogamy relations for entanglement negativity. Phys. Rev. Lett..

[49] He H, Vidal G (2015). Disentangling theorem and monogamy for entanglement negativity. Phys. Rev. A.

[50] Calabrese P, Cardy J, Tonni E (2012). Entanglement negativity in quantum field theory. Phys. Rev. Lett..

[51] Castelnovo C (2013). Negativity and topological order in the toric code. Phys. Rev. A.

[52] Lee YA, Vidal G (2013). Entanglement negativity and topological order. Phys. Rev. A.

[53] Lu H-X, Zhao J-Q, Wang X-Q, Cao L-Z (2011). Experimental demonstration of tripartite entanglement versus tripartite nonlocality in three-qubit greenberger-horne-zeilinger–class states. Phys. Rev. A.

[54] Wootters WK (1998). Entanglement of formation of an arbitrary state of two qubits. Phys. Rev. Lett..

[55] Osterloh A, Siewert J, Uhlmann A (2008). Tangles of superpositions and the convex-roof extension. Phys. Rev. A.

[56] Eltschka C, Siewert J (2012). Entanglement of three-qubit greenberger-horne-zeilinger–symmetric states. Phys. Rev. Lett..

[57] Siewert J, Eltschka C (2012). Quantifying tripartite entanglement of three-qubit generalized werner states. Phys. Rev. Lett..

[58] Horodecki M, Horodecki P, Horodecki R (1998). Mixed-state entanglement and distillation: Is there a “bound” entanglement in nature?. Phys. Rev. Lett..

[59] Żukowski M, Brukner Č, Laskowski W, Wieśniak M (2002). Do all pure entangled states violate bell’s inequalities for correlation functions?. Phys. Rev. Lett..

[60] Sen(De) A, Sen U, Wieśniak M, Kaszlikowski D, Żukowski M (2003). Multiqubit w states lead to stronger nonclassicality than greenberger-horne-zeilinger states. Phys. Rev. A.

[61] Chen K, Albeverio S, Fei S-M (2005). Concurrence of arbitrary dimensional bipartite quantum states. Phys. Rev. Lett..

[62] Verstraete F, Audenaert KMR, Dehaene J, Moor BD (2001). A comparison of the entanglement measures negativity and concurrence. J. Phys. A.

[63] Acín A (2000). Generalized schmidt decomposition and classification of three-quantum-bit states. Phys. Rev. Lett..

[64] Brun TA, Cohen O (2001). Parametrization and distillability of three-qubit entanglement. Phys. Lett. A.

[65] Cohen O, Brun TA (2000). Distillation of greenberger-horne-zeilinger states by selective information manipulation. Phys. Rev. Lett..

[66] Ajoy A, Rungta P (2010). Svetlichny’s inequality and genuine tripartite nonlocality in three-qubit pure states. Phys. Rev. A.

[67] Paul B, Mukherjee K, Sarkar D (2016). Nonlocality of three-qubit Greenberger-Horne-Zeilinger–Symmetric states. Phys. Rev. A.

